# The effect of AH26, Endoseal and ZOE sealers on Candida albicans and Enterococcus faecalis

**DOI:** 10.3205/dgkh000505

**Published:** 2024-10-23

**Authors:** Seyedeh Zahra Hosseini, Elham Aboualigalehdari, Mahnaz Fatahinia, Maryam Erfaninejad, Nahid Mahdian, Leila Gheitani, Reza Pakzad, Amin Kheiri

**Affiliations:** 1 Department of Periodontics, Faculty of Dentistry, Ilam University of Medical Sciences, Ilam, Iran; 2 Department of Parasitology and Mycology, Faculty of Paramedical Science, Ilam University of Medical Sciences, Ilam, Iran; 3 Department of Medical Mycology, School of Medicine, Ahvaz Jundishapur University of Medical Sciences, Ahvaz, Iran; 4 Department of Mycology, School of Medical Sciences, Shoushtar Faculty of Medical Sciences, Khuzestan, Iran; 5 Department of Microbiology, Faculty of Medicine, Ilam University of Medical Sciences, Ilam, Iran; 6 Department of Epidemiology, Faculty of Health, Ilam University of Medical Sciences, Ilam, Iran; 7 Student Research Committee, Ilam University of Medical Sciences, Ilam, Iran; 8 Department of Endodontics, Faculty of Dentistry, Ilam University of Medical Sciences, IIam, Iran

**Keywords:** endodontic sealer, antimicrobial, ZOE, AH26, Endoseal

## Abstract

**Background:**

In the oral cavity, root canal treatment failure (endodontics) is often due to the persistence of microorganisms in the root canal system after treatment, or re-infection of the root due to insufficient flooding. In addition, microorganisms are essential for the development of peri radicular diseases and are one of the main factors related to root canal treatment failure. Microorganisms that are commonly isolated from teeth that have failed endodontic treatment include *Enterococcus (E.) faecalis* and *Candida (C.) albicans*. Bacterial infection is closely related to the failure of root canal treatment, and the use of root sealer with antimicrobial activity and biological compatibility is necessary for the success of root canal treatment. One of the main goals of endodontic treatment is to eliminate intracanal infection and root canal flooding in order to prevent re-infection. Therefore, the aim of the current study is to evaluate the antibacterial and antifungal activity of ZOE, AH26 and Endoseal sealers *in vitro*.

**Methods:**

To study the effect of each sealer; AH26, ZOE and Endoseal on *E. faecalis* and *C. albicans*, 10 samples were considered. Firstly, the freshly prepared sealers were poured inside the microtube with nutrient broth and then 10 µl of *E. faecalis* and *C. albicans* suspensions were separately added into a microtube and were kept 24 hours in the incubator to grow.

**Results:**

The data were analyzed with Kolmogrov-Smirnov test and SPSS Version 19. Our results demonstrated that the resistance rate of *C. albicans* to ZOE, Endoseal and AH26 sealers was 0%, 100% and 70%, respectively. These values for the *E. faecalis* was 80%, 100% and 40% respectively. The resistance was defined when the microorganism was growth in subculture in LB agar.

**Conclusion:**

ZOE sealer has highest antimicrobial effect after that AH26 sealer and the least antimicrobial effect is related to Endoseal sealer.

## Zusammenfassung

**Hintergrund:**

In der Mundhöhle ist das Scheitern einer Wurzelkanalbehandlung häufig auf das Fortbestehen von Mikroorganismen im Wurzelkanalsystem nach der Behandlung oder auf eine erneute Infektion der Wurzel aufgrund einer unzureichenden Spülung zurückzuführen. Darüber hinaus sind Mikroorganismen für die Entwicklung periradikulärer Erkrankungen von wesentlicher Bedeutung und einer der Hauptfaktoren für das Scheitern von Wurzelkanalbehandlungen. Zu den Mikroorganismen, die häufig bei fehlgeschlagener endodontische Behandlung isoliert werden, gehören *Enterococcus (E.) faecalis* und *Candida (C.) albicans*. Da bakterielle Infektionen in engem Zusammenhang mit dem Scheitern einer Wurzelkanalbehandlung stehen, ist die Verwendung von Wurzelversiegelungen mit antimikrobieller Aktivität und biologischer Verträglichkeit für den Erfolg einer Wurzelkanalbehandlung unerlässlich. Eines der Hauptziele der endodontischen Behandlung ist die Beseitigung der Infektion im Wurzelkanal, um eine erneute Infektion zu verhindern. Ziel der vorliegenden Studie ist es daher, die antibakterielle und antimykotische Aktivität der Versiegelungen ZOE, AH26 und Endoseal *in vitro* zu untersuchen.

**Methode:**

Um die Wirkung der Versiegler AH26, ZOE und Endoseal auf *E. faecalis* und *C. albicans* zu untersuchen, wurden 10 Proben untersucht. Zuerst wurden die frisch zubereiteten Versiegelungen mit Nährbouillon in das Mikroröhrchen gegossen, dann wurden 10 µl einer Suspension von *E. faecalis* und *C. albicans* in das Mikroröhrchen gegeben und 24 h im Inkubator kultiviert.

**Ergebnisse:**

Die Daten wurden mit dem Kolmogrov-Smirnov-Test und der SPSS Version 19 mit folgendem Ergebnis ausgewertet. Die Resistenzrate von *C. albicans* betrug gegenüber ZOE, Endoseal und AH26 Versieglern 0%, 100% bzw. 70% betrug, von *E. faecalis* 80%, 100% bzw. 40%. Als Resistenz wurde definiert, wenn der Mikroorganismus in der Subkultur in LB-Agar wuchs.

**Schlussfolgerung:**

Die ZOE-Versiegelung hat die höchste antimikrobielle Wirkung, gefolgt von der AH26- und der Endoseal-Versiegelung.

## Introduction

In the oral cavity, the failure of root canal treatment (endodontics) is often due to the persistence of microorganisms in the root canal system after treatment or re-infection of the root canal due to insufficient flooding. In addition, microorganisms are responsible for the development of peri radicular diseases and are one of the main factors associated with root canal treatment failure. Ideally, for an optimal endodontic outcome, the bacterial and fungal populations within the root canal should be eliminated or significantly reduced to levels compatible with peri radicular tissue healing. If microorganisms remain after chemo mechanical preparation with or without intracanal medication, the risk of adverse consequences of endodontic treatment increases [1].

In fact, the presence of microorganisms such as bacteria and fungi in the root canal during filling has been shown to be a risk factor for apical periodontitis after treatment. Microorganisms that are commonly isolated from teeth that have failed root canal treatment include *Enterococcus (E.) faecalis* [2], [3], [4] and *Candida (C.) albicans* [5], [6].

The chronic failure of a root-treated tooth is due to the ability of *E. faecalis* to bind to the collagen of the dentinal tubule and survive inside the tubules. These microorganisms have the ability to grow even in low nutrient environments and can survive in root canals as mono infection [2]. *E. faecalis* has been detected in apical periodontitis lesions in root-treated teeth [3]. Facultative Gram-positive cocci of *E. faecalis* are present in more than one third of tooth canals with persistent periapical lesions [4].

*C. albicans* is a fungus that is often isolated from root infections. Although it is recognized by the dental pulp and peri radicular tissue cells that generate immune responses, it escapes the host's defenses and causes cell death. Then, the dentin tubule closes the tooth, forms a biofilm and attacks the dentin tubules to resist intracanal antiseptics and root canal treatments. Therefore, it is related to cases of persistent or resistant root infection. Fungi have been isolated from approximately 3 to 18% of infected root canals, with the predominance of Candida species, of which *C. albicans* is the most common fungus [5].

It has been shown that the microorganisms of teeth with unsuccessful endodontic treatment are different from what is usually found in untreated teeth [4]. In addition, it has been shown that root microorganisms have a high affinity for root canal filling materials, especially for gutta-percha. Due to the significant adhesion of bacteria, the subsequent biofilm formation can lead to the persistence of microorganisms in the root canal. It is well known that biofilms respond poorly to conventional antibiotics and may develop antibiotic resistance. In addition, the widespread use of antibiotics leads to the emergence of more resistant and dangerous species of microorganisms. As a result, the detection of new antimicrobial tools becomes very important for new treatment options [1].

Bacterial infection is closely related to root canal treatment failure, and the use of root sealer with antimicrobial activity and biological compatibility is necessary for root canal treatment success [7]. One of the main goals of endodontic treatment is to eliminate intracanal infection and root canal flooding in order to prevent reinfection [8]. None of the dental materials provide complete flooding with the cavity walls, and micron spaces always remain in the gap between the material and the cavity wall, through which microorganisms can penetrate [9]. Although chemical preparation significantly reduces the number of microorganisms, 40–60% of root canals still remain positive for the presence of bacteria. The remaining microorganisms are usually located in dentinal tubules, sub canals and apical branches.

Therefore, the healing of the periapical lesion is prevented, or the long-term success of the root canal treatment is reduced. Therefore, root canal flooding should bury these remaining bacteria and prevent their access to peri radicular tissues and block any other communication between the oral cavity and peri radicular tissues [8]. Since the root canal system is different in terms of anatomical features, including fins and straits and sub-canals, it is difficult to completely remove bacteria from the root canal, and infection may occur in canals with high anatomical complexity with a large number of facultative anaerobic bacteria. Flooding of the root canal space is necessary to bury any remaining bacteria and finally destroy them in the filled root canal [7].

One of the main goals of endodontic treatment is to eliminate intracanal infection and root canal flooding in order to prevent re-infection. Therefore, the aim of the current study is to evaluate the antimicrobial activity of AH26, ZOE and Endo sealers *in vitro*.

## Methods

### Ethical statement

The present study is an in vitro experimentation type. This study has been approved with the ethics ID IR.MEDILAM.REC.1401.066 and with the design ID A-10-3553-1 in Ilam University of Medical Sciences, Ilam, Iran.

This research was conducted on two types of microorganisms, *C. albicans* and *E. faecalis*. *C. albicans* isolates have been isolated from the oral mucosa of HIV-infected patients referred to the Behavioral Diseases Counseling Center of Jundishapur University of Medical Sciences, Ahvaz, Iran.

### Sealers

AH26 is an epoxy resin-based sealer, Endoseal is a calcium silicate and bioceramic based sealer and ZOE is a zinc oxide eugenol-based sealer. ZOE Product packaging included 20 gr powder, 10 ml liquid, 1X Dispensing Dropper, 1X spoon and 1X mixing pad; the powder contains Zinc oxide, barium sulphate, accelerators, and preservative; the liquid contains eugenol & excipients.

### Instructions for use

A scoop of powder and 3–5 drops of liquid to obtain smooth and homogenous mix, which can easily be introduced in to the canal. The dispensed material shall not be placed back in container and the dose once applied and used shall not be reused.

EndoSeal MTA included composition calcium silicates, calcium aluminates, calcium alumina ferrite, calcium sulfates, radiopacifier, thickening agent. It was premixed and pre-loaded in a syringe (no powder/liquid mixing required). 

AH2G Product packaging included 8 gr powder, 10 gr resin; the powder contains bismuth trioxide, calcium hydroxide, hexamethylenetetramine, and titanium dioxide; the liquid contains bisphenol-epoxy resin.

### Sample collection

For *C. albicans* isolates a wet cotton swab dipped in physiological serum was pulled firmly on the points of the oral mucosa where there were white lesions. To transfer the swab from the laboratory, a tube containing 0.5 ml of sterile physiological serum was used. Before providing the sample, the participation in the present research and the method of sampling were explained to the patient or his companion, and if the patient consented, the sampling was done. *E. faecalis* was collected from oral cavity. 

### Sample size

To calculate the sample size, the results of Arias-Moliz et al. [10] study were used, and the amount of colony forming units (cfu) per mL in the AH Plus sealer material was 153,000±110,000 and in the AH Plus + 1% bioceramic (BC) material it was considered as 94,000±225,000. Considering the first type error of 5% and power of 80%, the sample volume in each group was calculated based on the following formula of 10 samples. Since in this study we had 3 groups and 1 control group, the total number of samples (people in total) were 40 samples (Equation 1).

Equation 1:


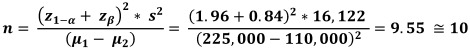


Inclusion criteria was patients that were selected based on having oral lesions (white pseudo membrane or whitish cream plaques), redness, inflammation, dry mouth, change in taste sensation and burning sensation during eating

After culturing the oral swab on Candida chrome agar culture medium, the number of cfu/ml was counted, the grown samples with the number of colonies ≤10 were included in the plan.

Confirmation of *C. albicans* samples was done based on molecular and macroscopic methods.

The experiment as done as below:

- First, all isolates of *C. albicans* and *E. faecalis* were cultured on sabro dextrose agar culture medium combined with chloramphenicol and blood agar and were incubated for 48 hours at 37°C and blood agar for 2 hours, respectively.
- In the next step, a suspension with a concentration of half McFarland was prepared from each of the cfu.
- Then, according to the instructions of the manufacturer, sealers were prepared.
- In the next step, 1 ml of each sealer was immediately added to the microtubes through the sampler and spread on their walls.
- In the next step, 10 ml of suspension and 1,490 ml of liquid culture medium (LB broth) were added to each of the mentioned microtubes.
- Then the microtubes were kept in an incubator at 37°C for 24 hours.
- In the next step, 10 ml of the solution inside the microtubes containing sealers and suspension of *C. albicans* and *E. faecalis* were transferred on Sabro dextrose agar culture medium.
- Then the plates were incubated at 37°C for 24 hours and the number of cfu on the culture medium was counted.

It should be noted that necessary controls including negative control (culture medium and sealer) and positive control (culture medium plus *C. albicans* and *E. faecalis*) were considered in this research and the samples and controls were done in triplicate. 

These procedures on blood agar were performed for *E. faecalis*. The non-growth colony on plate agar medium indicated as effectiveness of sealer.

### Statistical analysis

SPSS version 19 software was applied for data analysis. Normality of data was determined by Kolmogrov-Smirnov test. All quantitative data were presented as mean ± standard deviation if normal, otherwise logarithmic transformation was done for this default and qualitative data were reported as number (percentage). Independent t-test and analysis of variance were used to compare the mean in the studied groups, and if their defaults were not established, their non-parametric equivalents, i.e., Mann-Whitney and Kruskal-Wallis, were used. The significance level was 5%.

## Results

Our results demonstrated that the resistance rate of *C. albicans* to ZOE, Endoseal and AH26 sealers was 0%, 100% and 70%, respectively. These values for *E. faecalis* was 80%, 100% and 40% respectively (Table 1 (Tab. 1)). The resistance was defined when the microorganism was growth in subculture in LB agar.

**Figure 1:**
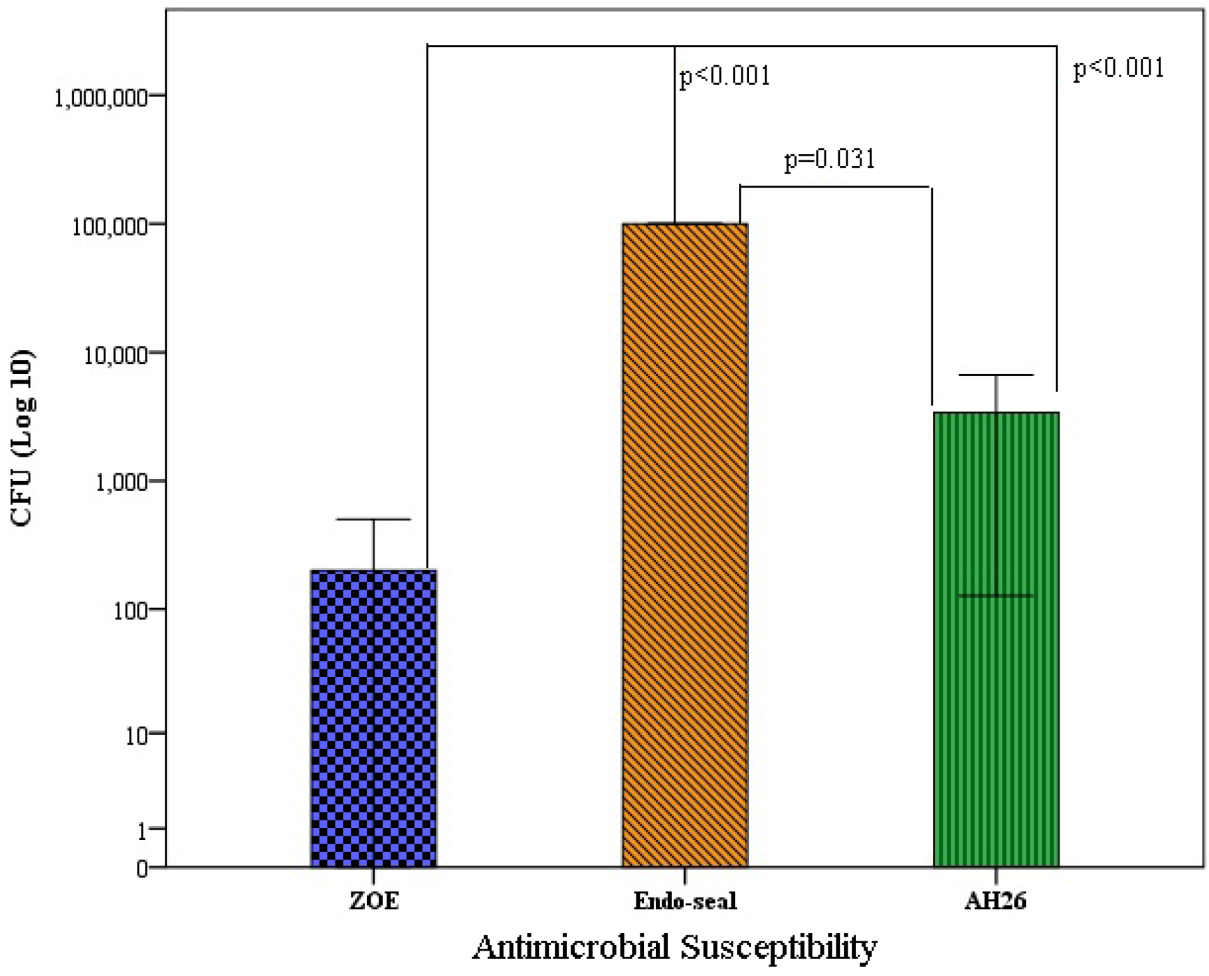
Average number of *E. faecalis* grown in each plate according to the type of sealer

In the positive growth of *C. albicans* and *E. faecalis*, we cultured the positive wells on blood agar and Saboraud dextrose agar. The results showed the average and standard deviation of the cfu per mL of *C. albicans* and *E. faecalis* grown in different root sealers. Therefore, the average number of cfu by *E. faecalis* in contact with ZOE sealer was 2×10^2^±421.63 cfu/mL, with Endo sealer was 100×10^3^±0.001 cfu/mL and for AH26 sealer was 34×10^2^±4575.29 cfu/mL. In other words, ZOE sealer had the highest antibacterial effect and Endoseal had the lowest antibacterial effect. Based on the results of the one-way analysis of variance test, there was a significant difference in the number of cfu by *E. faecalis* (P<0.001). This finding is also shown in Figure 1 (Fig. 1). Based on the results of Tukey’s post hoc test, the average cfu by *E. faecalis* in ZOE sealer were lower than Endoseal (P<0.001) and AH26 (P<0.001). Also, the average number of cfu formed in Endoseal was higher than AH26 (P<0.001).

**Figure 2:**
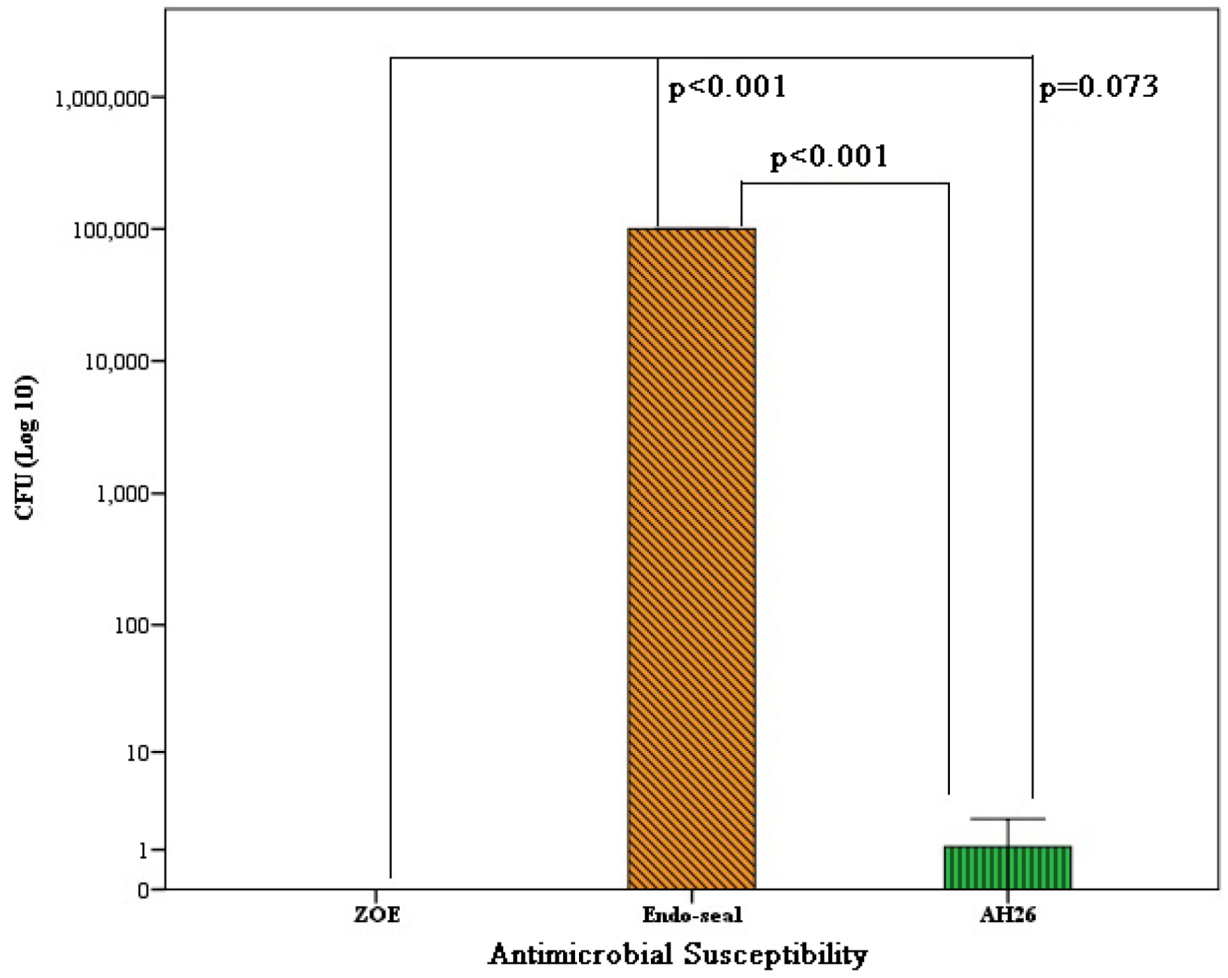
Average number of *C. albicans* grown in each plate according to the type of sealer

Also, our results showed the average number of *C. albicans* cfu in exposure to ZOE sealer was 0.01±0.01 CFU/mL, with Endo sealer was 100×10^3^±0.001 CFU/mL and with AH26 sealer was 1.1±1.85 CFU/mL. With other words, the highest antifungal effect was related to ZOE sealer and the lowest antifungal effect was related to Endoseal sealer. Based on the results of one-way analysis of variance, there was a significant difference in the cfu of *C. albicans* d (P<0.001). This finding is also showed in Figure 2 (Fig. 2). Based on the results of Tukey’s post hoc test, the average cfu of *C. albicans* in ZOE sealer was lower than that of Endoseal (P<0.001), but it was no significantly difference observed from AH26 (P=0.073). Also, the average cfu formed in Endoseal was higher than AH26 (P<0.001) (Figure 2 (Fig. 2)).

## Discussion

The antimicrobial activity of the three root canal sealers was evaluated against *E. faecalis* and *C. albicans*, which are associated with persistent periapical infection [4], [5]. Ideally, root sealers should be dimensionally stable and non-toxic. They should be able to create a strong bond with the dentin of the root canal to seal the walls well and prevent microleakage [11]. Also, it is desirable if the root sealers have strong and long-term antimicrobial effects effect. The additional antimicrobial effects of the root sealer will be beneficial in eliminating residual microorganisms that have survived the chemical and mechanical means of root canal treatment. As a result, the success rate of modern root canal treatment can be increased [12].

Sealers based on epoxy resin (AH26) have good antimicrobial, physical and chemical properties. Therefore, these sealers reduce the survival of microorganisms during obturation [13]. The calcium silicate-based sealer Endoseal was not introduced until 2014. Hence, studies investigating its antimicrobial activity are limited. Bioceramic sealers are known for their antimicrobial properties during setting and do not shrink [13]. The high antimicrobial activity of Endoseal can be due to a combination of high pH and active diffusion that increase the permeability of molecules such as calcium hydroxide in the cytoplasm of the bacterial cell and thus exert their antimicrobial effect [8]. Because of its alkaline pH, it helps to eliminate microorganisms such as *E. faecalis*, which do not survive in high pH, close to 11.5 or more [13]. 

The results of the present study showed a significant decrease in the number of microbes with ZOE sealer compared to AH26 and Endo- seal. ZOE showed maximum antimicrobial activity against *C. albicans* followed by *E. faecalis*.

Castillo-Villagomez et al. [14] tested AH26 (epoxy resin type) and Endoflas (ZOE). Their results with the agar diffusion method were that Endoflas was a stronger bacterial growth inhibitor than AH26, which is consistent with our results.

Rathod et al. [15] investigated the antimicrobial effect of Bioceramic and AH Plus sealers against *Staphylococcus aureus* and *C. albicans* and showed that AH Plus sealer has the most antimicrobial properties against *S. aureus* and *C. albicans* [15], which is consistent with the results of our study.

## Conclusions

ZOE showed the most micro biostatic efficacy against *E. faecalis* and *C. albicans*. AH26 and Endoseal had the lowest efficacy. Based on their long-term success, ZOE sealers have been recognized as a standard in endodontic treatment since their development. These sealers are still popular due to short sitting, low cost, antibacterial properties and ease of use [16].

### Limitations 

One limitation is the origin of the tested *C. albicans* strain from oral mucosa of HIV-infected patients instead of testing with a defined reference strain. The results are therefore only of limited value.

## Notes

### Authors’ ORCIDs

- Amin Kheiri: 0000-0002-6342-4818
- Elham Aboualigalehdari: 0000-0002-2517-8847
- Amin Fatahinia: 0000-0001-6898-1309
- Maryam Erfaninejad: 0000-0003-1071-9257
- Reza Pakzad: 0000-0001-8133-3664

### Competing interests

The authors declare that they have no competing interests.

### Shared first authorship

Seyedeh Zahra Hosseini and Elham Aboualigalehdari contributed equally to this work.

## Erratum

- Change of affiliation information for Kheiri A
- Change of affiliation information for Fatahinia M

## Figures and Tables

**Table 1:**
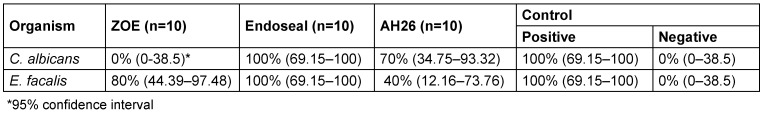
Percentage of* C. albicans* and *E. faecalis* growth in different root sealers
